# inTB - a data integration platform for molecular and clinical epidemiological analysis of tuberculosis

**DOI:** 10.1186/1471-2105-14-264

**Published:** 2013-08-30

**Authors:** Patrícia Soares, Renato J Alves, Ana B Abecasis, Carlos Penha-Gonçalves, M Gabriela M Gomes, José B Pereira-Leal

**Affiliations:** 1 Instituto Gulbenkian de Ciência, Rua da Quinta Grande 6, Apartado 14, Oeiras P-2781-901, Portugal; 2 Present address: Instituto de Higiene e Medicina Tropical, Lisbon, Portugal

## Abstract

**Background:**

Tuberculosis is currently the second highest cause of death from infectious diseases worldwide. The emergence of multi and extensive drug resistance is threatening to make tuberculosis incurable. There is growing evidence that the genetic diversity of *Mycobacterium tuberculosis* may have important clinical consequences. Therefore, combining genetic, clinical and socio-demographic data is critical to understand the epidemiology of this infectious disease, and how virulence and other phenotypic traits evolve over time. This requires dedicated bioinformatics platforms, capable of integrating and enabling analyses of this heterogeneous data.

**Results:**

We developed inTB, a web-based system for integrated warehousing and analysis of clinical, socio-demographic and molecular data for *Mycobacterium sp.* isolates. As a database it can organize and display data from any of the standard genotyping methods (SNP, MIRU-VNTR, RFLP and spoligotype), as well as an extensive array of clinical and socio-demographic variables that are used in multiple countries to characterize the disease. Through the inTB interface it is possible to insert and download data, browse the database and search specific parameters. New isolates are automatically classified into strains according to an internal reference, and data uploaded or typed in is checked for internal consistency. As an analysis framework, the system provides simple, point and click analysis tools that allow multiple types of data plotting, as well as simple ways to download data for external analysis. Individual trees for each genotyping method are available, as well as a super tree combining all of them. The integrative nature of inTB grants the user the ability to generate trees for filtered subsets of data crossing molecular and clinical/socio-demografic information. inTB is built on open source software, can be easily installed locally and easily adapted to other diseases. Its design allows for use by research laboratories, hospitals or public health authorities. The full source code as well as ready to use packages is available at http://www.evocell.org/inTB.

**Conclusions:**

To the best of our knowledge, this is the only system capable of integrating different types of molecular data with clinical and socio-demographic data, empowering researchers and clinicians with easy to use analysis tools that were not possible before.

## Background

Tuberculosis (TB) is usually a chronic, slowly progressing disease that frequently remains undiagnosed for many years. One-third of the world population is thought to be infected and in 2010 there were around 9 million new active cases of TB [1]. It is the second highest cause of death from an infectious disease worldwide, after HIV, and the biggest killer of people infected with HIV [2]. The rapid evolution of drug resistance strains is threatening to make TB incurable.

To control the progression of this disease, we need to define risk factors for transmission. To accomplish that, we need detailed clinical and socio-demographical information. In scenarios of intense transmission, it is essential to identify the source patient in order to prevent activation of recent infections. On the other hand, in communities where transmission is rare, the main goal would be to identify people who are latently infected, since most of the disease cases are a consequence of reactivated latent infection [3,4].

Another question that remains unanswered is whether specific characteristics are features of individual strains or broader strain lineages. Defining the nature of diversity in *M. tuberculosis* offers an ideal starting point for evaluating the clinical implications of such diversity [4-6]. The properties required to address the bacterial diversity are unlikely to be met by a single marker. Since standard sequence-based genotyping, such as Multilocus sequence typing (MLST) is not applicable in these bacteria, non sequence-based tools such as Variable Number Tandem Repeat (VNTR) based techniques have become the gold standard for routine genotyping and have been successfully applied to answer a variety of epidemiological questions [2,7-10]. While the significance of deep phylogenetic information for molecular epidemiology is yet to be established, unequivocal classification of bacterial strains is essential, in fact crucial if phenotypic associations are to be unveiled [6,7]. One way to address this problem is to combine different typing methods in order to take full advantage of their combined results. IS6110 RFLP, MIRU-VNTR and spoligotyping are methods that can be used for epidemiological purposes but, unlike SNPs, they do not provide a robust phylogenetic picture [11,12].

Addressing these questions requires an integrated framework, capable of linking clinical and socio-demographic data with molecular data. This framework should be able to read sequence data from bacterial isolates, identify global patterns and automatically classify strains into families [4,13]. Currently there are a few excellent public databases and web tools focused on tuberculosis. SpolDB4 [14] provides a clear picture of the current *M. tuberculosis* complex genome diversity, through Spoligotypes, with around 2000 sequences representative of several regions of the world. Nevertheless, it is not possible to correctly define the phylogenetic relationship of different strains only through Spoligotypes. MIRU-VNTRplus [15] and SITVIT [16] are broader than SpolDB4; they allow users to analyze and compare genotypes based on several methods: spoligotype, MIRU-VNTR, LSP, SNP or a combination of these markers. Although these databases contains information about sensitivity to drugs, little or no clinical data is available nor can it be uploaded, and without this information it is not possible to address the questions raised above.

Other existing approaches, not specific to tuberculosis, allow users to upload and analyze their data, such as MLST [17,18]. MLST is used by public health laboratories and researchers to query nucleotide data against databases over the Internet, but this system lacks clinical and/or socio-demographic information and does not provide any tools to analyze the data. Other systems have been designed for local installation, such as EpiPATH [19] developed as a generic framework for managing clinical and molecular data from infectious diseases. However, EpiPATH lacks any analysis tools, and requires programming-dependent customization to be used for a complex disease such as tuberculosis, with multiple typing methods and complex clinical data. Finally, generic systems like Bionumerics by Applied Maths NV. are widely used as data management and analysis tools, but they are commercial and costly.

While all the databases/platforms described above have their merits, none provides a means to locally integrate and analyze the complexity of tuberculosis within the context of a research, public health or clinical unit. In this work we describe a novel integrative framework, inTB, developed to fill this gap. It is a free, locally installable, customizable data management and analysis system for *Mycobacterium* disease, aimed at the research laboratories, public health authorities, and potentially for the clinical setting. inTB integrates different types of molecular data with clinical and socio-demographic information, and provides pre-defined data analysis and reporting tools. Adoption of this system ensures data consistency by use of validation mechanisms, and data reusability, by use of the provided analysis tools. inTB contrasts with existing dedicated databases and tools (see above) by providing local data management and analysis. It thus addresses privacy and confidentiality concerns by providing easy-to-use packages for local installation and use, without requiring that sensitive information is transmitted over the Internet. Furthermore, inTB brings to the fore extensive clinical and socio-demographic data that can be analyzed together with genotypic information, and should the user wish to do so, it is simple to expand to include more variables. InTB was designed bearing in mind both the needs of our collaborators at the National Tuberculosis Program in Portugal (Programa Nacional de Luta Contra a Tuberculose), a national public health authority, and our own needs as research laboratories investigating the molecular epidemiology of *M. tuberculosis*.

## Construction and content

### Database schema

**Figure 1:**
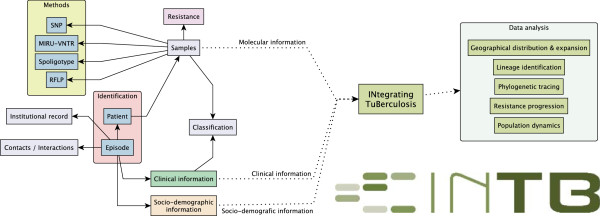
**Overview of inTB.** The system takes as input molecular typing, clinical and socio-demographic data that is stored in a relational database. inTB allows users to analyze their data, identify lineages and to export data for external analyses.

inTB was designed to integrate all major aspects of TB infection. This is reflected in the database schema, which consists of three major blocks: clinical, socio-demographic and molecular information. Each of these are linked via a clinical episode, which is the main entity of the schema, therefore it is extremely important to correctly understand the definition of ‘episode’. Episode refers to a single occurrence of tuberculosis illness. Each entry in the database corresponds to a diagnosis, which means that a patient can have more than one episode. If the patient has two occurrences at different times he will have two episodes that can have clinical and/or socio-demographic characteristics. The definition of ‘episode’ as the main unit was necessary to deal with cases of sequential occurrences of disease. Thus each individual case of tuberculosis can be associated with different clinical, socio-demographic or molecular information. Figure 1 represents the general schema of inTB.

The clinical part of the database stores information on BCG scar, vaccination and revaccination, diagnosis, symptoms, appointment date, number of previous regimens, toxicity, predicted period for completion of treatment, radiology, clinical form, and several others. While the socio-demographic part of the database has information on gender, country of origin, geographical localization, education and risk factors, such as, if the patient smokes, is drug dependent, alcoholic, unemployed, among others. An additional block for contact information include tables for the relationship with the contact (family member, neighbor, coworker, etc.) as well as a table for the symptoms.

On the molecular part of the database, there is a separate table for each of the typing methods: Spoligotype, SNP (information on the SNPs used can be found here: [20]). MIRU-VNTR and RFLP. An additional table stores the individual strain predictions for each of these genotyping methods, for each isolate, since there can be conflicts about lineage identification between different methods (see below). A separate table is used to associate each sample to the episode it was identified in. Tables for first and second line drug resistance are available, including tables with the antibiotics used. Additional file 1 represents the complete database schema for all tables.

Each episode can have more than one sample, which allows for the identification of possible co-infections, but a single sample cannot belong to a different patient. The episode table is used to traverse all relationships between the three different blocks of the database. In case one of the blocks of information is not provided, the system is still able to perform correctly. The stored information is displayed via a web interface, either textual or graphically (described below).

## Implementation

inTB is written in Python 2.6.5, can be installed on UNIX/Linux systems and virtualized on Microsoft Windows and Mac OSX. To store the information, a MySQL server version 5.1 was used. The system was implemented on Django 1.4 and runs on Apache with mod_WSGI. Phylogenetic trees were built with BioPython 1.54 and NetworkX 0.99.

Access to inTB is made via a web browser. This choice removes the need for any additional software installation as all systems have a working web browser. Additionally, it gives the user the possibility of using either as a local system (local database), but also to be used with a centralized server with multiple distributed clients. inTB is compatible with most common browsers. The oldest recommended versions that were successfully tested are Mozilla Firefox 3.6, Apple Safari 5.1, Microsoft Internet Explorer 9 and Google Chrome 18. Newer versions were also tested successfully. As long as browsers remain compliant with HTML and CSS2/3 standards, future versions should work without problems. To enable the application’s full functional capability JavaScript must be enabled.

To facilitate the use of inTB and avoid installation problems, we provide a pre-built virtual machine to be executed in the VirtualBox platform. The user has only to follow some simple steps and s/he is able to access the data through the web interface. For non-programmer users this is an advantage because no programming is needed. The virtual machine allows users to run their own versions of inTB on their computers without the need to code anything. Once the system is locally installed, no additional connection to the Internet is necessary.

## Utility and discussion

### Data input

inTB was initially built for our own use (bioinformatics, genetics and epidemiology groups) and that of our collaborators (public health authorities, clinical groups). We made most of the features accessible via a graphical interface. This interface provides two ways to insert data; manual insertion via a form, or by uploading a comma separated value (CSV) file. Additionally, users may also insert data directly into the database by writing custom scripts. inTB includes verifications of variable type and term matching to minimize errors when uploading data. Detailed instructions on how to upload data are given in the manual, but a few points merit mention here. The first is that inTB automatically creates an episode number for each patient when new data is inserted. Since each patient has a unique identifier, this means that inTB can track multiple episodes per patient. The second is that a patient identifier is not required for molecular and resistance data, only a sample code is required. This enables the asynchronous uploading of data that can later be linked, when further data becomes available. The third point is that when adding new variables to the database, inTB encourages standards compliance, connecting the user to BioPortal [21] - a list of BioPortal terms used inTB is provided in Additional file 2. Finally, in its current implementation, inTB will integrate four types of genotypic data. However, inTB does not process as yet raw genotypic information for any method, which need to be transformed into a text file externally (SNP letter/positions, MIRU numbers, spoligotype and RFLP binary patterns).

inTB may be the first data management solution that a given user will have, or it will be used alongside existing data management solutions. In the latter case, data transfer solutions between existing systems and inTB will need to be implemented. Laboratories that generate molecular typing information on a routine basis will very likely have some sort of LIMS (Laboratory Information Management Systems) from which the molecular data will need to be exported to be uploaded to inTB. It may be used alongside or instead existing databases in public health laboratories/authorities. In our experience, dealing with data management systems from the Portuguese public health authorities and the Genotyping Laboratory at the Instituto Gulbenkian de Ciência, we solved this problem by implementing a semi-automated exporting of spreadsheets and simple scripts to format the data and upload it to inTB. Another problem that the user may experience is having data types that are not present in inTB. Since inTB is built upon open access tools, adding additional fields to the database is feasible and simple, but it will require some programing knowledge.

### Data access

**Figure 2:**
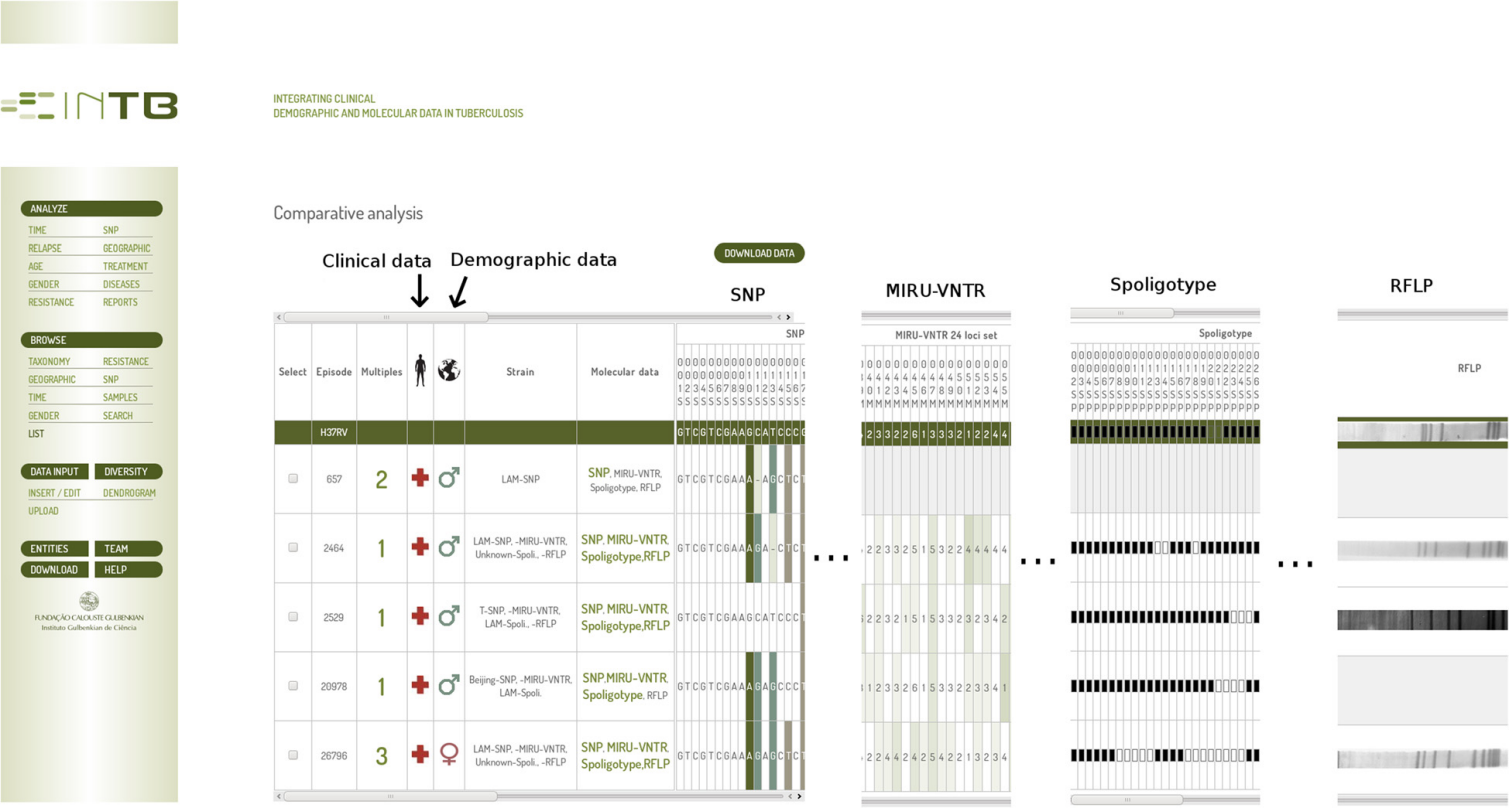
**Results page.** It can be accessed via the browse or search functions, or as in the case shown, for a subset of isolates that were selected by the user.

There are two major ways to access data via the web interface: browsing the content and searching for particular episodes, filtering by one of several criteria. Both methods result in the same output, the results page (Figure 2). This gives an overview of the most relevant information for each patient, and is likely of interest to researchers wishing to find trends in data. The user can download the data for external analysis.

The focus on patient was designed with clinical use at heart. It displays information about the current other episode of disease, follow-up and contact. The latter was included at the requests of clinicians, wishing to have the means to rapidly contact patients following a diagnosis of the disease, in order to to prevent other infections or quickly diagnose other patients. The inclusion follow-up information allows the monitoring of the progress of the status of the patient, allowing for quick diagnosis of possible reinfections, decreasing the risk of death and transmission in the community. Different episodes may be chosen for comparison of clinical and socio-demographic information, side by side. As before, when episodes are selected, it is possible to download all the information associated (clinical, socio-demographic and molecular).

### Data analysis

One of the novel features of this database, compared to existing resources, are the analysis tools provided. Within the analysis menu, the user can have a global overview of the data in the database, plotted according to different criteria. Every time new data is entered into the system, all the plots and dendograms are automatically updated. The user may also select a subset of data and/or variables for plotting. Moreover the user can download the raw data used to create each result in case they want to perform their own analysis with another method. Several analyses are implemented in the inTB interface, four of which we illustrate below, with an emphasis on phylogenetic analysis.

**Figure 3:**
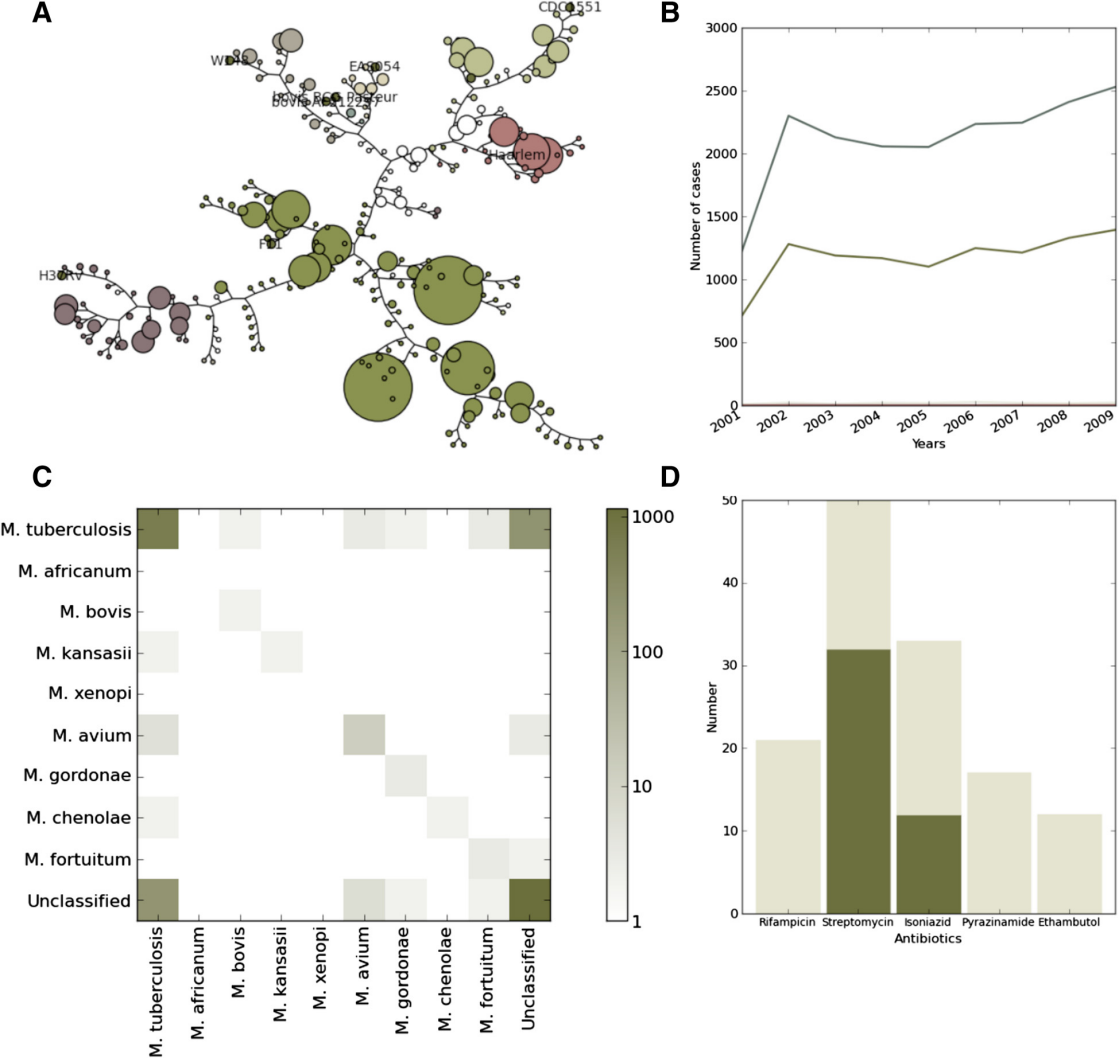
**Screenshots of some analyses features offered by inTB. (A)** SNP-based dendogram of strains in database **(B)** Infections over time, by species - the two lines represent unclassified (top) and M. tuberculosis (middle), with all other species close to or at zero **(C)** Recurrence matrix by species highlighting the rare occurrences of reinfections **(D)** Frequency of antibiotic resistances per SNP (SNP shown is in the gene rpoB, which is known to be involved in resistance to Rifampicin - dark green represent the reference allele).

inTB can generate and display phylogenetic trees, and it automatically classifies new isolates added to the database into pre-defined tuberculosis families. We compiled SNPs and specific spoligotype patterns from the literature allowing to unambiguously identify the lineages [14,22-24]. As soon as new molecular data is uploaded into inTB, the system will automatically align the samples, build a tree and color each sample according to a specific lineage. inTB builds a maximum likelihood tree, using PhyML [25], for SNPs, based on an alignment performed with Mauve [26]. For the three other methods, inTB calculates a Neighbor-Joining tree, based on a distance matrix calculated according to the Manhattan distance (MIRU-VNTR) or Hamming distance (RFLP/Spoligotype). The phylogenetic tree for RFLP was built through a binary file exported from Bionumerics [27]. Note that if the user wants to use another phylogenetic method, the alignment is provided for download. We show in Figure 3A the output of a SNP analysis, and it reveals for example that the LAM lineage dominates the population in the database, followed by Harlem, and that while the latter is not very diverse, the former shows considerable diversification. The user is given also the option of generating trees based on a subset of the data (*e.g*. all the isolates from 2010 onwards).

Tuberculosis typing methods have varied over time, and new methods tend to supersede older ones. This means that for older data we may have a dominance of one method (RFLP, Spoligotype), but for new isolates MIRUs and SNPs may dominate. To enable users to deal with this data heterogeneity, inTB can also calculate supertrees. A supertree is a phylogenetic tree assembled from a combination of smaller phylogenetic trees. By combining the phylogenetic tree obtained by SNPs, MIRUs, Spoligotype and RFLP, a single, comprehensive tree called supertree is created. This supertree is built with Matrix Representation Parsimony (MRP). This method converts each tree into a matrix of binary characters. All the matrices are then combined into a single matrix, with question marks in any cell for a sample not found in a particular clade. The parsimony tree is the supertree. Note that the use of supertrees is controversial [28,29].

In Figure 3B we show a temporal analysis of all species. Other variables could be plotted over time such as the incidence of drug resistance. Figure 3C shows a heatmap comparing recurrence patterns - if a patient has a recurrence, this heatmap allows reveals whether they are by the same or a different species. SNPs involved in the development of resistance are powerful markers for detection of first and second line resistance. Users can generate bar charts of SNP frequency, for example as a function of drug resistance (Figure 3D).

All the analysis and variability features are available online. However, we considered that our target audience needs to have the results in different formats - to present results in conferences or seminars, or as recurrent reports in case of public health laboratories/clinical centers. We have thus implemented the option of creating pre-defined reports that generates tables and charts in either a PDF or a CSV format. We pre-defined five types of reports as example: (i) variation of gender and resistance over time, (ii) occurrence of new cases, (iii) re-treatment over time, (iv) proportion of episodes occurring in immigrants, with information of the country of origin, as well as the (v) percentage of missing data, for each variable, according to the selected year. The user is able to select the variable of interest and generate a PDF or CSV report.

### Testing/usability

An empirical usability test was performed in order to to determine whether the design and tools implemented allowed users to manage information easily through the interface, and to identify the main difficulties while navigating through the website. The test involved 16 participants with different backgrounds, as given on Table 1. We used time to complete each task as the metric in this test. Subjects read a short tutorial and were then asked to complete several tasks on a test database (Additional file 3) - the time that each task took to complete was recorded for each participant independently. One of the authors also did the test to give us a reference (shortest) time. The main results are summarized in Table 2. Overall the users did not have problems in quickly solving the problems in the test, nor did they take much time to do so, considering that for most of them it was the first contact with the website. In all the cases where users took a bit longer or reported a higher difficulty were simplified as per user’s suggestions. Note that this is a second test, as earlier in the development phase we conducted a smaller heuristic evaluation [30] of inTB to identify problems and avenues for improvement - user’s suggestions at that stage were particularly important when implementing the final design of the site.

**Table 1:** Summary of the characteristics of the participants in the usability test

**Characteristics**	**Value**
Number of participants	16
Average age	34 years
Academic studies	43.75% bioinformatics,
	18,75% maths,
	12.5% biochemistry,
	6.25% computer science,
	6.25% medicine,
	6.25% biology.
Gender	62.5% Male, 37.5% Female
Previous experience with databases	75% Yes, 25% No
Experience with tuberculosis	32% Yes, 68% No

**Table 2:** **Summary of the results of the usability test, for the test population shown in Table**1**, and for one of the developers (PS), given as reference time**

**Task**	**Average time, in seconds**	**Reference time, in seconds**
1	41.1 (17.9 – 90.6)	4.2
2	82.2 (57.3 – 113.3)	22.7
3	76.7 (17.5 – 215)	9
4	68.6 (20–130)	18
5	108.9 (38–270)	23
6	28.6 (6.7 – 48.7)	6.2
7	74.5 (15–155)	-

Five tasks were considered: (1) Browsing through the data, (2) Entering data into the system, (3) Searching specific episodes, (4) Updating records, (5) Generate reports, (6) Analyze several graphics and (7) locally install inTB and access it.

## Conclusions

inTB is an open source information system for storing, managing and analyzing data from clinical and molecular information on tuberculosis, which is available for download and local installation. This system allows identification of lineages of *M. tuberculosis* strains based on different genotyping methods. Furthermore inTB combines this information with clinical and socio-demographic information, allowing new types of analyses. inTB was conceived to be used both in the research laboratory, and by public health or clinical centers. It is currently used within a research setting at the Instituto Gulbenkian de Ciência, in a collaborative project with public health authorities in Portugal. Its adaptation to other infectious diseases is being discussed with the Portuguese National Health Institute. We believe that inTB fills the gap of a free software that can simultaneously store and analyze epidemiological data for tuberculosis, for use by researchers, clinicians and public health authorities, and that provides both the easy to use web-based interface for the non-programers, as well as the normal programatic access of open access platforms.

## Availability and requirements

A demo version of inTB is available at http://www.evocell.org/inTB. At the same address an installation version, and a virtual machine image, can be downloaded. inTB runs on Unix/Linux and can be virtualized in Mac OSX and Windows systems. It has been tested in a variety of browsers (Mozilla Firefox 3.6, Apple Safari 5.1, Microsoft Internet Explorer 9 and Google Chrome 18). inTB is distributed under a BSD 3-clause license.

## Abbreviations

TB: Tuberculosis; SNP: Single nucleotide polymorphisms; MIRU: Mycobacterial interspersed repetitive units; RFL: Restriction fragment length polymorphisms; HIV: Human immunodeficiency virus; LSP: Large sequence polymorphisms; BCG: Bacillus Calmette-Guérin.

## Competing interests

The authors declare that they have no competing interests.

## Authors’ contributions

MGMG and JPL conceived and supervised the project; CPG provided data and discussed the results, PS, ABA and RJA implemented the system; PS and JPL wrote the manuscript. All authors read and approved the final manuscript.

## Supplementary Material

Additional file 1 Detailed inTB DB schema. Click here for file

Additional file 2 Summary of ontology terms used. Click here for file

Additional file 3 inTB usability test. Click here for file
