# Supplementary figures and images for: inTB - a data integration platform for molecular and clinical epidemiological analysis of tuberculosis
Source: BMC Bioinformatics. 2013 Aug 30;14:264. doi: 10.1186/1471-2105-14-264 (PMC3847221; doi:10.1186/1471-2105-14-264)

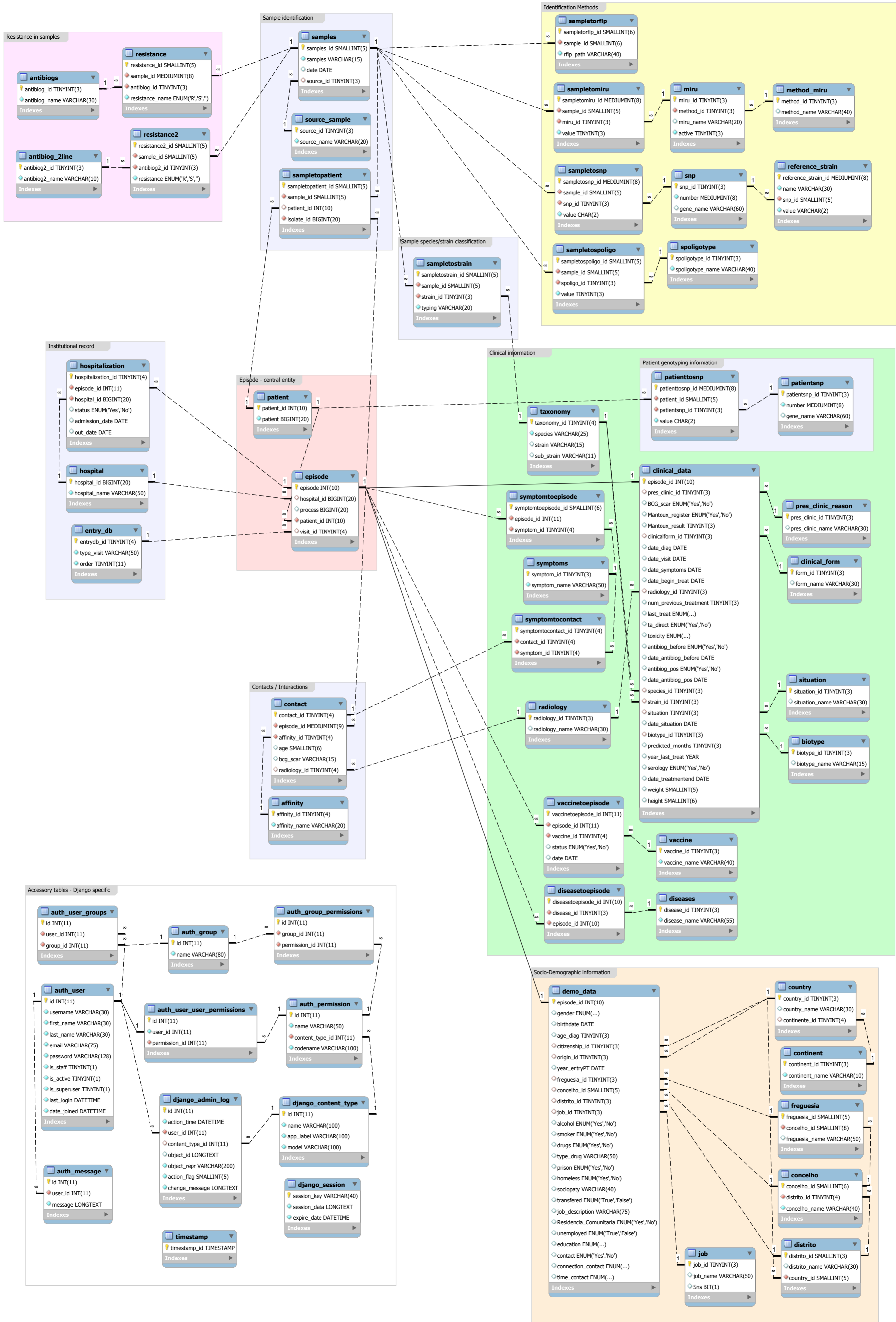

Supplement: Additional file 1 — Detailed inTB DB schema. [file 1471-2105-14-264-S1.pdf]
