# Supplementary material for: inTB - a data integration platform for molecular and clinical epidemiological analysis of tuberculosis
Source: BMC Bioinformatics. 2013 Aug 30;14:264. doi: 10.1186/1471-2105-14-264 (PMC3847221; doi:10.1186/1471-2105-14-264)
Supplement: Additional file 3 — inTB usability test. [file 1471-2105-14-264-S3.pdf]

Usability Test

Introduction and content description

InTB was created to help organize and link clinical, demographic and different molecular data of portuguese tuberculosis patients.

In the system, each patient can have one or more records of infections, named here as episodes. Each episode can have one or more samples which may have been analyzed with different methods (SNP, MIRU-VNTR, Spoligotype, RFLP).

To access the data, the user may browse episodes through the menu on the left. It's possible to filter results by taxonomy, geographical location, year of diagnosis, gender, resistance to antibiotics or browse all the genotyped samples.

Alternatively one may search for episodes with specific characteristics. This option allows the user to customize their search for different and/or multiple parameters.

The output of searching or browsing will always be a table where each row is one episode. By hovering or clicking each cell more information is displayed.
The column “Multiple” represents how many infections a patient had (number) and how many samples were collected (color – green for 1 sample, pale-yellow more than 1). When clicked, the full patient history will be displayed, including clinical, socio-demographic, molecular, resistance, contact information as well as follow-up and missing information.
The “Molecular data” column displays methods which are currently stored in inTB. If the method has information, its name will be displayed in green. When clicked the table expands showing the relevant details.
For detailed information about each column, please refer to the User Manual.

While navigating results the user can select individual episodes for later comparison. Simply check the box in the first column and click the add button at the bottom of the table. When enough episodes are selected a message will show up at the bottom of the screen with instructions.

If the user wishes to add information to inTB s/he can do so in two ways. For small datasets or to correct existing data one can use the Data Input forms. For bulk insertion the file Upload approach is preferred. The User Manual provides detailed information on each option.

A distinctive feature of inTB is the ability to perform predefined analysis on user submitted or existing data. One can see the variation of species/strains in relation to time, gender, age or geographical regions. compare reinfection rates between different species, treatment abandonment rates or variation of resistance through time.
In all cases the user can download a digested file with the raw data and recreate the plots with their preferred tools.

Finally and as a way to summarize data, the user can select between a predefined set of analysis to generate a report based on diagnostic year. These can be found under the Analysis section of the menu.

Advanced users can also have their own local installation of inTB by navigating to the Download section and following the provided instructions.

NOTE: Descriptions/definitions of underlined terms are available in the Glossary on the last page

Tasks

Task 1 - Browse data

1 Find all episodes of infection in males
  1.1 How many of these are lacking molecular information?
  1.2 On the first episode of the list, how old was the male at the time of infection?
  1.3 On the same episode, what Clinical form was identified?
  1.4 Have any patients listed in the first page recurred?
2 Find all episodes of infection by *M. tuberculosis Haarlem*
  2.1 How many patients were infected by this strain?
  2.2 On the first episode of the list, find if the patient had a BCG scar?
  2.3 Do all episodes in the first page have the same genotype (visually compare SNP)?

Task 2 - Insert data

3 Insert one patient episode via the available forms (National patient ID is 50000).
  3.1 Clinical data:
    - Patient displays **Pulmonary** form of infection by ***M. tuberculosis LAM***.
  3.2 Demographic data:
    - Patient was born in **Bangladesh** is currently **homeless**, **smokes** and has a **drug addiction**.
  3.3 Molecular data
    - Add one sample (with ID **50000-A**) to same patient where SNP[1] 2284 was called as a **T.**

Task 3 - Search data

4 Search for the patient you just inserted.
5 Identify which fields are lacking information.

Task 4 - Update data

6 Update the same patient by adding the **Date of birth** – 1st March 1975.

Task 5 - Generate reports

7 Generate a report with the number cases of patients born in France, Germany and United Kingdom diagnosed between 2008 and 2010.

Task 6 - Analyze results

8 What is the age distribution of patients infected by the *LAM* strain?
9 What is the geographical distribution of *M. tuberculosis LAM* by district?
10 Is resistance to the antibiotic Streptomycin becoming a bigger concern?
11 How frequent are re-infections by a different species/strain from the first infection?

Task 7 - Install locally

12 Locate the instructions for local installation.
  12.1 Follow the quick install instructions
  12.2 Open browser pointing to local installation

---

1 In real scenarios, more SNPs would be analyzed but it would be too time consuming to insert them manually. Instead they would be inserted using the CSV file method.

Glossary

Episode – Record of infection. One patient may have more than one episode if infection reoccurs by the same or a different species/strain.

Sample – Biological sample which is then analyzed with different methods. More than one sample may be collected for a single episode.

SNP – Single Nucleotide Polymorphism, identifying single nucleotide variations scattered across the genome.

MIRU-VNTR - Mycobacterial Interspersed Repetitive Units - Variable Number Tandem Repeat, a method by which you can count the number of times a sequence is repeated in a given set of locus.

RFLP - Restriction fragment length polymorphism, a method based on the restriction pattern of a predefined set of enzymes.

Spoligotype – a method which detects presence or absence of 43 unique spacers between repeats in a specific locus.

Clinical form – different symptoms which reflect the type of tuberculosis infection (e.g. Pulmonary, Meningitis,...).

H37RV – a strain of *M. tuberculosis* commonly used as reference.

User Manual – can be found by clicking the HELP button on the left-menu or on the Download section of the inTB website.
